# Emergence times and airway reactions in general laryngeal mask airway anesthesia: study protocol for a randomized controlled trial

**DOI:** 10.1186/s13063-015-0855-2

**Published:** 2015-07-26

**Authors:** Ana Stevanovic, Rolf Rossaint, András P. Keszei, Harald Fritz, Gebhard Fröba, Friedrich Pühringer, Mark Coburn

**Affiliations:** Department of Anesthesiology, University Hospital RWTH Aachen, Pauwelsstr. 30, 52074 Aachen, Germany; Department of Medical Informatics, University Hospital RWTH Aachen, Pauwelsstr. 30, 52074 Aachen, Germany; Department of Anesthesiology and Intensive Care Medicine, Martha-Maria Hospital, Röntgenstraße 1, 06120 Halle (Saale), Germany; Department of Anesthesiology, University Hospital Ulm, Albert-Einstein-Allee 23, 89081 Ulm, Germany; Department of Anesthesiology and Intensive Care Medicine, Klinikum am Steinenberg, Steinenbergstr. 31, 72764 Reutlingen, Germany

**Keywords:** laryngeal mask airway, emergence time, airway reaction, anesthesia

## Abstract

**Background:**

The use of a laryngeal mask airway (LMA) in appropriate patients supports fast-track anesthesia with a lower incidence of postoperative airway-connected adverse events. Data on the most favorable anesthetic in this context, with the lowest rate of upper airway complications and fast emergence times, are controversial and limited. Desflurane seems to match these criteria best, but large randomized controlled trials (RCTs) with a standardized study protocol are lacking. Therefore, we aim to compare desflurane with other commonly used anesthetics, sevoflurane and propofol, in a sufficiently powered RCT. We hypothesize that desflurane is noninferior regarding the frequency of upper airway events and superior regarding the emergence times to sevoflurane and propofol.

**Methods/Design:**

A total of 351 patients undergoing surgery with an LMA will be included in this prospective, randomized, double-blind controlled, multicenter clinical trial. The patients will be randomly assigned to the three treatment arms: desflurane (*n* = 117), sevoflurane (*n* = 117), and propofol (*n* = 117). The emergence time (time to state the date of birth) will be the primary endpoint of this study. The secondary endpoints include further emergence times, such as time to open eyes, to remove LMA, to respond to command and to state name. Additionally, we will determine the frequency of cough and laryngospasm, measured intraoperatively and at emergence. We will assess the postoperative recovery on the first postoperative day via the Postoperative Quality Recovery Scale.

**Discussion:**

Despite increasing importance of cost-effective and safe anesthesia application, we lack proof for the most advantageous anesthetic agent, when an LMA is used. There are only a few RCTs comparing desflurane to other commonly used anesthetics (sevoflurane, propofol and isoflurane) in patients with LMA. These RCTs were conducted with small sample sizes, huge interstudy variability, and some also showed strong biases. The present multicenter RCT will provide results from a large sample size with a standardized study protocol and minimized bias, which is feasible in the clinical routine. Furthermore, we will expand our knowledge regarding the most favorable recovery on the first postoperative day, which impacts patients’ comfort after surgery.

**Trial registration:**

EudraCT Identifier: 2014-003810-96, 5 September 2014

ClinicalTrials.gov: NCT02322502, December 2014

**Electronic supplementary material:**

The online version of this article (doi:10.1186/s13063-015-0855-2) contains supplementary material, which is available to authorized users.

## Background

### Background and rationale

Fundamental pillars of anesthesia care are the safe and measured application of anesthesia. Improvement of facility and resource utilization with a high patient turnover and early discharge of patients from the operating room and hospital is becoming increasingly important. This hinges on a rapid emergence and early recovery from anesthesia. Desflurane’s low blood gas coefficient supports general anesthesia (GA) in a fast-track technique, even in obese patients [1]. The use of a laryngeal mask airway (LMA) attenuates the risk of postoperative airway-connected complications in comparison to endotracheal tubes (ETT) in GA [2].

Desflurane’s possible airway irritant properties compared to other anesthetics like sevoflurane, propofol and isoflurane are still the subject of discussions [1]. However, studies comparing the risk of intraoperative upper airway adverse events like cough and laryngospasm (LS) vis-à-vis other common anesthetics are limited. One meta-analysis [3] considered these adverse events in patients with LMA and could not evince a greater incidence with desflurane compared only to sevoflurane anesthesia. Data regarding recovery times were not assessed in this meta-analysis. Therefore, we have recently performed a further meta-analysis [4] comparing desflurane’s properties to other commonly used anesthetics with LMA: isoflurane, sevoflurane and propofol. We identified only 13 randomized controlled trials that have analyzed the variables of upper airway adverse events (intraoperative cough (CO), cough at emergence (CE) and laryngospasm (LS)) and the recovery times (time to open eyes (TOE), to remove LMA (TLR), to respond to command (TRC) and time to state date of birth (TSB)). While the frequency of upper airway adverse events did not differ between the analyzed anesthetics, the recovery times in the desflurane groups were superior to the other anesthetic groups. The variable CO showed a non-significant RR (95 % CI) of 1.12 (0.63, 2.02), (*P* = 0.70), between desflurane (*n* = 284) and all other examined anesthetic agents (*n* = 313). CE was only examined after desflurane (*n* = 148) and sevoflurane (*n* = 146) administration and also indicated no difference, RR [95 % CI] of 1.49 (0.55, 4.02), (*P* = 0.43). LS was rare and desflurane (*n* = 262) showed no difference when compared to all other analyzed anesthetics (*n* = 289), or when compared only to sevoflurane, RR (95 % CI) of 1.03 (0.33, 3.20), (*P* = 0.96). All emergence-time variables were significantly faster in the desflurane group. Due to the small sample sizes, huge study protocol distinctions and the fact that the examined variables were mainly secondary endpoints of the included studies, these findings have to be seen within their limits. Additional large randomized controlled trials are indicated to validate the results of our meta-analysis [5].

### Objectives and study design

This will be a multicenter, controlled, double-blind, randomized, three-arm parallel, interventional clinical study. The purpose of this study will be to assess the following:

1. If desflurane is superior in achieving a faster emergence from anesthesia (when patient can state his/her date of birth).
2. If desflurane is noninferior in the occurrence of upper airway complications compared to sevoflurane or total intravenous anesthesia with propofol in the setting of laryngeal mask airway.

### Specific primary objective

The specific primary objective is to determine the time of emergence from anesthesia with LMA (defined as time between cessation of anesthesia until patient is able to state his/her date of birth (TSB) on command) after desflurane compared to sevoflurane or propofol.

### Specific secondary objectives

The specific secondary objectives are to determine the frequency of airway reactions (CO, LS, laryngospasm at emergence (LSE) and CE) in patients undergoing desflurane anesthesia with LMA, compared to sevoflurane or propofol. Additionally, we will analyze further emergence times (TLR, TOE, TRC, time to state the full name (TSN) on command and the recovery index [6]) in the same patients after cessation of anesthesia.

### Other secondary objectives

Other secondary objectives are to determine the influence of gender on the patients’ reactions, the anesthesia safety variables including hemodynamic stability, the frequency and quantity of postoperative nausea, vomiting and pain, the discharge times, the pharmacoeconomics and the recovery on the first postoperative day (1^st^ POD).

## Methods/Design

### Study setting

This multicenter study will be conducted in four German study centers (University hospitals of Aachen and Ulm and the regional hospitals of Reutlingen and Halle).

### Eligibility criteria

Investigators in each center will be selected and supervised by the local coordinating principal investigator. To ensure the blinding process, at least two investigators will be needed, when a patient is enrolled in this study. Therefore the screening and enrollment process will be performed according to the logistical possibilities of each center. All potential patients meeting the inclusion criteria will be documented in a screening log kept at each site. The reasons for not enrolling eligible patients will have to be stated in the screening log.

Inclusion criteria: A written informed consent prior to study participation will be mandatory. Participants in the study have to be adult patients between 18 and 75 years of age. They have to be scheduled to undergo elective surgery with a planned duration of 0.5 to 2 hours and the use of LMA. Both sexes will be included, and further inclusion criteria are an American Society of Anesthesiologists (ASA) physical status 1 to 3 and body mass index (BMI) <35 kg m^−2^.

Exclusion criteria: Subjects fulfilling one or more of the following exclusion criteria will not be included in the study: patients with planned additional regional and local anesthesia, with asthma or chronic obstructive pulmonary disease (COPD) grade IV, contraindication for the use of a LMA, known allergy or hypersensitivity to any drugs administered during this study. Furthermore, women who are pregnant, breast-feeding or of childbearing potential and not using adequate contraceptive methods will be excluded. Patients legally unable to give written informed consent, nonfluency in German language, with severe psychiatric or neuropsychiatric disorders or recent (<6 months) history of alcohol or drug abuse will not be included. The participation in a drug or device trial within the previous 30 days will be a further exclusion criterion.

### General interventions for all patients

Premedication: All patients will receive an oral administration of up to 7.5 mg midazolam 30 to 45 minutes preoperatively, according to the clinical routine of each center.

Induction: Upon arrival into the surgery room, a standard monitoring will be applied to all patients. This will consist of continuous electrocardiogram (ECG), measurement of non-invasive blood pressure (NIBP) and peripheral oxygen saturation (SpO_2_). A connection of Bispectral Index (BIS) monitoring will follow. After pre-oxygenation with oxygen (FiO_2_ = 1.0) for 3 minutes via face-mask, all patients will receive remifentanil injection via infusion pump at an initial rate of 0.5 μg kg^−1^ over 60 seconds and 1.5 to 2.5 mg kg^−1^ (plus 2 ml lidocaine 1 %) propofol by bolus titration intravenous (i.v.). After loss of consciousness, the LMA will be inserted and blocked with a cuff pressure at 30 cm H_2_O. Teeth marks on the device will help to check the correct insertion. The leakage will have to be controlled by applying a positive airway pressure of 25 cm H_2_O. A reassessment will take place after 5 minutes. If the LMA is not tight, a replacement of LMA will follow. After a second failure of LMA placement, an alternative airway will be applied.

Maintenance: All patients will receive a postoperative nausea and vomiting (PONV) prophylaxis with 8 mg dexamethasone and 4 mg ondansetron, if the Apfel-Score is ≥2. Analgesia will be maintained with remifentanil injection via infusion pump at a rate of 0.15 μg kg^−1^ min^−1^. Dosage will be adapted according to clinical needs based on the patient’s hemodynamic (difference of NIBP and HR greater than 20 % from baseline), autonomic (sweating, salivation, or flushing) and somatic (movement or swallowing) signs. Twenty minutes before the estimated end of surgery, all patients will receive 0.05 to 0.1 mg kg^−1^ piritramide and 15 mg kg^−1^ metamizole i.v. The inspired oxygen concentration will be adjusted to 35 to 50 %, or above this range if medically indicated. The end-expiratory CO_2_ level during the maintenance of anesthesia should be kept between 36 and 45 mmHg. The use of muscle relaxants is undesired, except for emergency situations. Spontaneous breathing or controlled ventilation with LMA will be applied during maintenance of anesthesia, according to the standard operating procedure of each participating center. Inspired desflurane concentrations of >8 vol. % and sevoflurane >2.2 vol. % have to be avoided. The systolic blood pressure should be kept at ≥90 mmHg (or ≥65 mmHg for mean arterial blood pressure), and a relative variation > 20 % from baseline values has to be avoided and requires appropriate treatment. If indicated additional bolus injection of propofol is permissible in patients.

End of anesthesia: At five minutes before planned termination of surgery, remifentanil will be discontinued. The LMA will be removed when the upper airway reflexes are fully recovered and the respiratory function is adequate (regular spontaneous breathing with a frequency of at least eight breaths per minute and SaO_2_ >95 %) and when the patient opens his/her eyes on request or is able to follow any other requests. In addition, the patient has to be hemodynamically stable before removal of LMA. The patients will be admitted to the Post Anesthesia Care Unit (PACU) following surgery. They will receive 0.05 mg kg^−1^ piritramide if the visual analog scale (VAS) for pain is more than 30.

### Specific interventions

There will be three parallel intervention groups receiving different anesthetics for the maintenance of anesthesia after the induction phase and placement of the LMA.

Group 1 will receive desflurane. After setting the fresh gas flow at 2l min^−1^, the desflurane vapor will be turned to 12 vol. % until the desired end-expiratory target concentration of 0.8 MAC (minimal alveolar concentration) or 4 to 5 vol. % desflurane is achieved. Thereafter, a reduction of the fresh gas flow to 500 to 1,000 ml will be performed to maintain anesthesia. Desflurane concentration will be adjusted to maintain a BIS index value between 40 and 60. Five minutes before estimated termination of surgery, the vapor will be set to zero. At the end of the surgery maximum fresh gas flow will be applied. This time-point will be defined as time zero (T_0_).

Group 2 will receive sevoflurane. After setting the fresh gas flow at 2l min^−1^, the sevoflurane vapor will be turned to 8 vol. % until the desired end-expiratory target concentration of 0.8 MAC or 1.2 to 1.4 vol. % sevoflurane is achieved. Thereafter, a reduction of the fresh gas flow to 500-1000ml will be performed to maintain anesthesia. Sevoflurane concentration will be adjusted to maintain a BIS index value between 40 and 60. Five minutes before estimated termination of surgery, the vapor will be set to zero. At the end of the surgery maximum fresh gas flow will be applied. This time-point will be defined as time zero (T_0_).

Group 3 will receive propofol via infusion pump at an initial rate of 5 to 7 mg kg^−1^ h^−1^ propofol and then adjusted to maintain a BIS index value between 40 and 60. Five minutes before estimated termination of surgery the propofol concentration will be halved. Propofol will subsequently be discontinued and maximum fresh gas flow with F_i_O_2_ = 1.0 will be applied at the end of surgery. This time-point will be defined as time zero (T_0_).

### Interventions - modifications

All drugs used in this trial are anesthetics used daily in the clinical routine of all participating centers. Harms are not anticipated in any study treatment. All possible side effects are described in the summary of medicinal product characteristics (SmPC). Other potential side effects and changes in the frequency of possible side effects are not expected during the trial. A modification or discontinuation of the assigned study intervention is not expected. In the event of modifications or discontinuations of the study treatment, study participants will be retained in the study to enable data collection and preclude missing data.

### Outcomes

#### Primary outcome measures

The primary objective of this trial is to investigate, whether the emergence time until the stating of the date of birth after desflurane anesthesia is superior to the time after sevoflurane or propofol anesthesia. The selection of stating the date of birth as the primary clinical outcome variable is associated with the result of our latest meta-analysis [4]. This meta-analysis supposes faster emergence times in the desflurane-treated patients, but the included studies had only small sample sizes ≤65 patients per group. Furthermore, they were not all powered for this outcome variable, and the study protocols were not standardized for all patients. The time to state the date of birth was chosen, since this variable requires the most conscious answer compared to the other emergence variables. This primary outcome time variable is defined as time between cessation of anesthesia (T_0_) until the patient is able to state his/her date of birth on command (given every 20 sec.) and will be measured in minutes and seconds.

#### Secondary outcome measures

The secondary outcome measures will be the determination of the following additional emergence times:

- Time to remove laryngeal mask (TLR)
- Time to open eyes on command (TOE)
- Time to respond on command to press hand (TRC)
- Time to state the full name on command (TSN)
- Recovery Index RI = 1 + Aldrete_5 min/[(2 x extubation time) + 1 x opening eyes time)]_ [6]

These secondary outcome variables will also be assessed in minutes and seconds after cessation of anesthesia (T_0_). Furthermore, we aim to analyze the frequency of upper airway events in desflurane-anesthetized patients compared to sevoflurane and propofol. These variables were also analyzed in our meta-analysis [4] and indicated that there were no distinctions between the anesthetics. According to the emergence times, the included studies had only small sample sizes and were not all powered for these variables. Our study is also powered to show a noninferiority of intraoperative coughs in desflurane versus sevoflurane and propofol anesthetized patients. The following upper airway events will be assessed:

- Frequency of intraoperative coughs, CO (induction/maintenance) - noninferiority design
- Frequency of intraoperative laryngospasm, LS (induction/maintenance)
- Frequency of cough at emergence, CE
- Frequency of laryngospasm at emergence, LSE

#### Other outcome measures

Other outcome measures will include the following:

- Sex effects
- Measuring depth of anesthesia (BIS)
- Hemodynamic parameters (NIBP/Heart Rate (HR))
- Requirement of catecholamines
- Respiratory parameters (Airway pressures and end-tidal carbon dioxide: CO_2_)
- Modified Aldrete score
- VAS pain in the PACU
- Nausea in the PACU
- Frequency of vomiting in the PACU
- Time to readiness to be discharged from PACU
- Pharmacoeconomics
- Postoperative Quality Recovery Scale (PQRS) including the recovery on 1^st^ POD

### Participant timeline

A participant chart is shown in Additional file 1. Eligible patients, scheduled for surgery, will be presented to the anesthesia department for the general anesthesia consent preoperatively. Additionally, a blinded sub-investigator will explain the nature and purpose of the study to these patients and seek a written informed consent for the study (time point A, before surgery). The same investigator will collect the following baseline characteristics after the patient enrollment (time point A):

- Age
- Sex
- Weight
- Height
- BMI
- Smoking status (nonsmoker, current smoker-amount of pack years, ex-smoker)
- Contraceptives (woman - yes/no)
- Pre-existing diseases and medical/surgical history
- Classification according to the American Society of Anesthesiologists (ASA)
- Apfel Score
- Baseline (Postoperative Quality Recovery Scale) PQRS testing prior to surgery (see Additional file 2)

Shortly before surgery on the same day (time point B), an unblinded investigator will randomize the patients with sequentially numbered, opaque, sealed envelopes (SNOSE).

The same unblinded investigator will perform anesthesia (time point C, surgery) and assess the intraoperative variables in the respective patient:

- Depth of anesthesia (BIS)
- Standard safety parameters: ECG, SpO_2_ and end-tidal CO_2_, and NIBP
- Ventilation pressure parameters: Ppeak, Pmean, and PEEP
- Type of performed surgery
- Recording of the end-expiratory applied volatile anesthetic concentration/applied amount of propofol for maintenance
- Pharmacoeconomics: Total amount of intraoperative required catecholamines, of additionally applied propofol as rescue medication, of wasted propofol at the end of the surgery in the propofol group and of intraoperative remifentanil and piritramide consumption.
- Time points of anesthesia induction, LMA insertion, administration of the study treatments, anesthesia and surgery duration
- Frequency of intraoperative (induction/maintenance) coughs and laryngospasms

The blinded investigator will enter the operating room at the time point T_0_, which starts with cessation of anesthesia and application of maximum fresh gas flow and predefines the measurement start-time point for the time variables. The unblinded investigator will ensure that the blinded individual is precluded from recognizing which anesthetic was used, by masking the ventilator. The blinded investigator will assess the following postoperative variables during time point D:

Assessment of airway reactions

- Frequency of CE and LSE
- Emergence times: TSB, TLR, TOE, TRC, TSN
- The modified Aldrete scores with a 10-point scale will be recorded 5 minutes after removal of LMA (see Additional file 3)

Time point E starts with admission of the patient to the PACU. The blinded investigator will assess the following variables:

- The modified Aldrete scores with a 10-point scale will be recorded every 15 minutes until discharge from the PACU (see Additional file 3)
- Time-point of readiness to be discharged from the PACU (Aldrete Score ≥9) (see Additional file 3)
- Assessment PQRS-T40, which is 40 minutes after the time point T_0_ (see Additional file 2).
- The VAS pain score will be self-evaluated and recorded by 100-mm visual analog scale (VAS) (with 0 corresponding to no pain and 100 corresponding to maximal pain) every 15 minutes after admission to the PACU. For postoperative analgesia, the patients will receive 0.05 mg kg^−1^ piritramide, in order to maintain a VAS pain score ≤30 mm

Other measurements in the PACU (blinded assessor) include the following:

- Nausea in the PACU will be evaluated using an 11-point verbal rating scale (VRS) (with 0 corresponding to no nausea and 10 corresponding to extreme nausea) at admission and discharge from the PACU and every 15 minutes in the PACU
- Count of vomiting in the PACU
- Consumption of piritramide in the PACU

Time point E: First post-op day: (telephone interview/ visit at ward):

- PQRS-D1 (see Additional file 2)

### Sample size

Sample-size estimates for both considerations (superiority and noninferiority) were calculated separately. All sample sizes were calculated using a type 1 error, α = 0.05, and power of 0.80. The primary outcome calculations were carried out based on a one-way analysis of variance (same n) with three groups. The variances and means were estimated for the primary outcome variable time to state the date of birth from our meta-analysis [4]. For the sevoflurane, propofol and desflurane groups, means were set to 8.75, 6.8 and 5.6 minutes, respectively. A standard deviation of 3 minutes was considered (these parameters correspond to an effect size (f) of 0.43). A sample size of 19 patients per group is needed to detect such an effect for our superiority hypothesis.

The sample size for the noninferiority hypothesis was calculated based on the secondary outcome variable cough overall (CO). Using the normal approximation to the binomial and assuming a proportion of the outcome in the population between 0.07 and 0.10 and noninferiority bound of 0.20 a sample size of 81 and 112, respectively, is required to claim noninferiority. The margin delta of 20 % was chosen for clinical considerations. We claim that a difference of cough occurrence up to 20 % is clinically not relevant. This is justified by the result of our meta-analysis [4], where the identified coughs had never lead to a serious impact on the patients’ outcomes.

The sample size calculation for noninferiority of treatment is based on a sample size formula published by Blackwelder [7]. Considering data from the literature, a dropout rate of 5 patients per group (for example, requirement of endotracheal intubation, protocol deviations and losses for first postoperative day- follow up) is expected. We decided therefore to include 117 patients per group, to cover the calculated sample size of 19 + 5 patients per group for the superiority hypothesis as well as 112 + 5 patients for the noninferiority hypothesis. Altogether, there will be 351 randomized patients.

### Recruitment

Each participating center will recruit in 5 months as many patients as possible according to their clinical routine during the preoperative anesthesia consultation. Before inclusion of study patients, each center must ensure that at least two investigators are available: one physician for the randomization and the intraoperative visit and the second one for the pre- and postoperative visit. The exact time point of the informed consent has to be documented in written form in the patient source data to enable reproducing the sequence of patient recruitment and randomization, in order to prevent selection bias.

### Randomization

The responsible biostatistician will perform a study-specific computer-generated randomization (study arms, number of blocks and block lengths) according to the study-specific biometric specifications, by using the software framework “R” [8]. The complete randomization list and potential block sizes will only be accessible by the biostatistician of the study. To guarantee adequate allocation concealment, the group assignments will be preserved in sequentially numbered, sealed, opaque envelopes and these will be provided to the principle investigator of each center at the beginning of the study by the biostatistician. The principle investigator will ensure that only the unblinded investigators have access to these envelopes and that they will be opened in sequential order of the patients’ surgery time points. Additionally, he will have to assure that the anesthesia is truly performed according to the assigned intervention, to avoid performance bias. In order to maintain blinding during enrollment and data entry to the electronic case report form (eCRF), two different accounts will be created per center. By utilizing the first user account, access to intraoperative data, as well as randomization, will be possible. Using the second account, entry of pre- and postoperative data will be permitted. Patients in compliance with all inclusion criteria and none of the exclusion criteria will be randomly assigned into one of the three study groups: desflurane, or sevoflurane, or total intravenous anesthesia with propofol.

Access to randomization is reserved solely to the intraoperative investigator and has to be done shortly before surgery. The time point of envelope opening, the patient initials and the screening number of the patient will be written on the appropriate envelope before it is opened. The envelopes have to be safely stored in the intraoperative patient file. Within a center, the randomization number will occur in succession. The number will contain information about the randomizing study site and the patient sequence. The randomization number will consist of six digits, the first three for the center and the last three for the patient, for example, 001 to 001 (for center 001 and the first patient included). This randomization code will be used to label all information collected for each patient. The monitors of the study will check carefully that the envelopes were really used in the correct sequence and that the signed informed consent occurred definitively before opening of the randomization envelope.

### Blinding

The trial participant and the investigator performing the pre- and postoperative visits will remain blinded throughout the entire study period. The unblinded intraoperative investigator will ensure that the blinded individual is prevented from recognizing which anesthetic was used, by masking the ventilator with an opaque board before the blinded investigator enters the operating room. The blinded investigator will not have access to the anesthesia records until the study participation of each patient is completed.

### Unblinding procedures

In the event of medical emergency that requires identification of an individual patient’s treatment, blinded investigators will be allowed to contact the unblinded investigator or peruse the anesthesia protocols. The reason must be documented in the patient’s medical record and eCRF and must explain why revealing the treatment assignment was essential to guide subsequent intervention and therapy.

### Data collection methods

The coordinating principal investigator will ensure local training to enhance the data collection quality and reduce bias. The investigator will ensure that all assisting study personnel will be adequately qualified and informed about the study protocol, any amendments, study medication and study related responsibilities and functions. The investigator will maintain a study staff authorization log.

Each center will provide a stopwatch with minutes and seconds to measure awaking times. The modified Aldrete Score and PQRS form are enclosed in the Additional file 2 and 3 of the study protocol. After an initial pilot study of 133 patients, the PQRS tool was revised to its current form, and a validation study of 701 participants was conducted [9]. The VAS and VRS scales are valid, reliable and appropriate for use in clinical practice [10]. Once a patient is enrolled or randomized, the study site will make every reasonable effort to follow the patient for the entire study period until completion of phase G.

### Premature treatment termination

The study will be terminated prematurely (dropout) for an individual subject if the following occurs: serious intraoperative anesthesia complication, with required postoperative transfer to intensive care unit for anesthesiological reasons. The time point and specific reason for premature treatment termination of each subject have to be documented. The investigator shall determine a fundamental reason for premature treatment termination of each subject. All relevant safety data until subject’s treatment termination will be collected and reported. The study will be terminated in the event the risk-benefit-ratio alters in such a way, that premature treatment termination is indicated to protect a subject’s health.

### Premature study termination

Participants may withdraw from the study for any reason at any time. As the follow-up period in this study is only one day, we do not expect many withdrawals. These withdrawals could only imply missing data for the PQRS testing at T40 and on 1^st^ POD.

### Data management

Standardization procedures will be implemented to ensure accurate, consistent, complete and reliable data, including methods to ensure standardization among sites (for example, training, newsletters, investigator meetings, monitoring, centralized evaluations, and validation methods). The monitors will be trained during a kick-off meeting. Preparatory training to prepare the investigators and to standardize performance will be held during an investigators’ meeting before study start. This study will be monitored regularly by a qualified monitor from the sponsor Clinical Trial Center Aachen (CTC-A) according to the good clinical practice (GCP) guidelines and the respective SOPs (see Section Monitoring).

All data collected from a subject during the course of this clinical study have to be entered and/or filed in the respective subject file (CRF, case report forms). These data will be considered as source data. The subject file must contain a detailed statement on the informed consent procedure. The subject’s participation in this study must be appropriately documented in the subject file with study number, subject number, date of subject information and informed consent, date of each visit, and date of a potential telephone contact. If a study site is using an electronic system for documenting source data, a member of the site staff must print out the source data after each visit. The paper printouts must be overlapping, if possible (for example, must contain at least the last row of data from the subject’s previous visit). Otherwise, the thoroughness of source data must be ensured by other suitable means. The printout must be signed and dated by a member of the site staff who can confirm the accuracy and completeness of data in the paper printout. Additionally, the monitor shall sign and date the verified paper printout. The paper printout shall be stored in the investigator site file (ISF). If the source data information is entered retrospectively, it must be done directly on the paper printout and must be initialed and dated. The same applies to any corrections of initial data. Paper printouts are not required if the site is using a validated computer system, including audit trail, with separate access for the monitor (for example, monitor has solely access to data of the study subjects). If corrections are necessary, the subject shall be instructed to draw a single line through the error, leaving the incorrect entry legible. The subject should date the correction, but not initial it. The investigator shall not falsify the documents. The sponsor’s data management function will be responsible for data processing in accordance with the sponsor’s data management procedures. Database lock will occur only after quality assurance procedures have been completed.

All source data related to study data should be kept in locked cabinets. Access to the study data will be restricted. Investigators will enter the information required by protocol into an electronic data collection system via internet (eCRF). The study data manager will develop the eCRF. Detailed information on the eCRF completion will be provided during the site initiation visits. An eCRF completion manual will be provided to each site. An e-learning tool will train all persons who enter data into the eCRF. After the successful completion of the training, all participants will receive a training certificate. The access to the e-learning tool and to the eCRF will be password-controlled. Plausibility checks (including valid values, range checks, and consistency checks) will be performed according to a data validation plan to ensure correctness and completeness of these data. Inconsistencies will be queried via the electronic data collection system; answers to queries or changes of the data will directly be documented in the system. The database will be closed after all data are entered and all queries are solved. By signing the CRF (eCRF/eSignature), the investigator will confirm that all investigations have been completed and conducted in compliance with the clinical study protocol, and that reliable and complete data have been entered into the eCRF. The investigator will keep the subject files and original data as long as possible and according to the local procedure, but at least for 10 years, as specified in the International Conference on Harmonisation, (ICH)-GCP Guideline. The investigator/institution should take measures to prevent accidental or premature destruction of these documents.

### Statistical methods

All efficacy, safety and economical results will be presented on the Intention to Treat (ITT) data set, which will be composed of all randomized patients. In case it appears necessary, we will define a secondary Per Protocol (PP) data set prior to the database lock. The PP would be a subset of the ITT data set, composed of all randomized patients who have no major protocol deviations throughout their entire study period. This PP data set would be used for the secondary supportive analysis of the primary efficacy criterion and serve as a check of its robustness.

Parametric data will be analyzed with ANOVA and nonparametric data with the two-tailed Fisher’s exact test if incidence <5 or the Chi-square test with incidence >5. The inference for the noninferiority will be based on the upper confidence bound. A fully specified Statistical Analysis Plan will be made public before unmasking results. Additional analyses are not planned.

### Data monitoring

The investigator is obliged to permit study specific monitoring, auditing and inspections by the competent ethics committee, to enable direct access to source data and source documents and to support the respective person to the best of his/her knowledge.

This study will be monitored regularly by a qualified monitor from the sponsor CTC-A according to GCP guidelines and the respective SOPs. Monitoring procedures will include one or more visits designed to clarify all prerequisites before the study commences. Interim monitoring visits will take place on a regular basis according to a mutually agreed upon schedule. During these visits, the monitor will check for complete entries in the eCRF/CRF: for compliance with the clinical study protocol, ICH-GCP principles, the Declaration of Helsinki, regulatory authority requirements, for the integrity of the source data with the eCRF/CRF entries, and for subject eligibility. Monitoring will also be used to detect any misconduct or fraud. In addition, the monitor will check whether all adverse events (AEs) and serious adverse events (SAEs) have been reported appropriately within the required time periods. The investigator and all staff will be expected to cooperate with the monitor by providing any missing information whenever possible. The investigator must be available to answer questions arising during regular monitoring visits. Additionally, the investigator is required to:

- Have all data recorded in the eCRF and subject files properly, prior to each monitoring visit
- Have the source documentation available at the monitoring visits

All subjects who give their informed consent, including those screened but not enrolled into the study, will be listed on the subject screening/enrollment log. Further details of monitoring activities will be set forth in the monitoring manual.

Interim analyses are not planned. Failure of this study is unlikely, as all study treatments are performed during clinical routine and the required patient number will probably be achieved during the clinical routine of the participating sites. If unexpected SAEs occur in connection with the study treatment, the coordinating principal investigator and the sponsor will discuss the termination of the trial.

### Harms

Safety assessments will consist of monitoring and recording all AEs and SAEs by regularly monitoring of the measured vital parameters and physical examinations. The investigator will be provided with AE and SAE reporting forms by CTC-A, receive training for AE/SAE definition, documentation and reporting. AE and SAE documentation and reporting will be monitored on site.

Definition of AEs:

An AE is defined in the ICH Guideline for GCP as “any untoward medical occurrence, including an exacerbation of a pre-existing condition, in a patient or clinical investigation subject administered a pharmaceutical product and that does not necessarily have a causal relationship with this treatment.” (ICH E6:1.2) Each AE has to be reported on an Adverse Event Case Report Form. As far as possible, each AE has to be described according to the following:

1. Its duration (start and end dates)
2. Its severity grade (mild, moderate, or severe)
3. Its relationship to the study drug (suspected/not suspected)
4. Treatment required and action taken with trial drug
5. Outcome
6. Seriousness

Examples of the severity grade, relationship to study treatment and actions taken, as presented in the case report form, are provided below. The severity grade of an AE provides a qualitative assessment of the extent or intensity of an AE, as determined by the investigator or as reported by the subject. The severity grade does not reflect the clinical seriousness of the event, only the degree or extent of the affliction or occurrence (for example, severe nausea, mild seizure), and does not reflect the relationship to study drug.

Severity grade for an AE:

The intensity of the AE should be judged based on the following:

1 = Mild: Awareness of sign(s) or symptom(s) that is/are easily tolerated
2 = Moderate: Enough discomfort to cause interference with usual activity
3 = Severe: Incapacitating or causing inability to work or to perform usual activities

Causal relationship of AE:

Medical judgment should determine the relationship, considering all relevant factors, including the pattern of reaction, temporal relationship, de-challenge or re-challenge, confounding factors such as concomitant medication, concomitant diseases and relevant history. An assessment of causal relationship must be recorded for each AE.

Causality will be reported as either “Yes” or “No”:

Yes: There is a reasonable causal relationship between the investigational product administered and the AE.
No: There is no reasonable causal relationship between the investigational product administered and the AE.

Definition of SAEs:

A serious adverse event (SAE) is defined as an adverse event as follows:

- Results in death (fatal)
- Is immediately life-threatening
- Results in persistent or significant disability/incapacity;
- Requires or prolongs patient hospitalization;
- Is a congenital anomaly/ birth defect;

or

- Based upon appropriate medical judgment, may jeopardize the patient and may require medical or surgical intervention to prevent one of the afore-mentioned outcomes (for example, intensive treatment in an emergency room without hospitalization)

A hospitalization meeting the regulatory definition for “serious” is any inpatient hospital admission that includes a minimum of an overnight stay in a health care facility. Any AE that does not meet one of the definitions of serious (for example, emergency room visit, outpatient surgery, or urgent investigation) may be considered by the investigator to meet the “other significant medical hazard” criterion for classification as a SAE. An event does not need to be reported as an SAE if it represents only a relapse or an expected change or progression of the condition that was the cause of treatment without any other symptoms and signs than those present before treatment. This type of event needs only to be reported as an AE.

Reporting Procedures of SAEs:

All SAEs have to be reported by the principal investigator to the sponsor (CTC-A) within 24 hours of discovery or notification of the event. The sponsor will collect all SAE reports and write the annual safety report.

### Auditing

Audits will be performed according to the corresponding audit program, including the possibility that a member of the sponsor’s quality assurance function may arrange to visit the investigator in order to audit the performance of the study at the study site, as well as all study documents originating there. Auditors conduct their work independently of the clinical study and its performance. Contract auditors may also perform audits. In that case, the sponsor’s quality assurance function will agree with the contract auditor regarding the timing and extent of the audit(s). In case of audits at the investigational site, the monitor will usually accompany the auditor(s).

### Ethics and dissemination

#### Research ethics approval

The study will be performed in accordance with the ethical principles that have their origin in the Declaration of Helsinki. In accordance with the Declaration of Helsinki, the German Medicines Act (Deutsches Arzneimittelgesetz (AMG)), as well as the GCP-Guideline, the study was presented to the leading Ethics Committee of the University of RWTH Aachen, 52074 Aachen, which is related to the coordinating investigator. The local Ethics Committees of the other three centers (1. Halle: Ethik-Kommission des Landes Sachsen-Anhalt, 06846 Dessau-Roßlau; 2. Reutlingen: Ethik-Kommission bei der Landesärztekammer Baden-Württemberg, 70597 Stuttgart; 3. Ulm: Ethics Committee of the University of Ulm, 89081 Ulm) had only advisory function. The ethical approval with the reference number EK 314/14 was received on 15 January 2015. Any change in the study protocol and/or informed consent form will be presented to the Ethics Committees. These would have to be approved by the leading Ethics Committee before implementation (except for changes in logistics and administration or when necessary to eliminate immediate hazards). Additionally, the sponsor had already requested approval from the respective competent authority (Federal Institute for Drugs and Medical Devices (BfArM), Germany). This request was approved on 15.12.2014. The sponsor will provide copies of the approval documentation to the principal investigator of each center for his/her files. Any change in the study protocol will have to be approved by the competent Authority. They have to be approved by the competent authority before implementation (except for changes in logistics and administration or to eliminate immediate hazards).

The notification of the clinical trial according to § 67 German Medical Act to the local supervising authority was performed by the sponsor according to the SOP and with the consent of the principal investigator. The same procedure will account for any amendments after the end of the study as well. The sponsor will provide a copy of the notification to the principal investigator for his files.

### Protocol amendments

The authorization of the relevant competent authority and approval of the ethics committee for any amendments that may become necessary during the study will be applied by the sponsor, CTC-A, according to the SOPs.

Reportable amendments are changes, which may affect the following:

- Safety of subjects
- Integrity and credibility of data
- Protocol amendment
- Changes in risk evaluation of drugs consisting/including genetically modified organisms

Every amendment of the protocol has to be signed by the coordinating investigator, the sponsor and the biostatistician.

### Consent

According to AMG and GCP-Guidelines, informed consent has to be obtained from subjects prior to participation in the trial. The subjects will voluntarily confirm their willingness to participate in the trial, after having been informed by a physician verbally and in writing of all aspects of the trial that are relevant to the subject’s decision to participate. Patients will be informed about the requirements concerning data protection and have to agree to direct access to their individual data. The subjects will sign an informed consent form for study participation as well as the disclosure of individual data. The informed consent form has to be signed and dated by the subject and one of the sub-investigators. Before written informed consent is obtained, the investigator must provide the subject ample time and opportunity to inquire about details of the trial and to decide whether or not to participate. All questions regarding the trial have to be answered to the satisfaction of the subject. The subject information and informed consent form will be prepared and informed consent will be obtained from the subject according to sponsors’ SOPs. The participants will receive copies of the informed consent forms. The participant will be informed by a physician in a timely manner if new information becomes available that may be relevant to the subject’s willingness to continue participation in the trial. The communication of this information will be documented. The subject will receive a copy of any amendments to the written information and a copy of the signed and dated consent form updates. Subjects will be informed that they are free to withdraw from the study at any time at their own discretion without necessarily giving reasons. The participation in the clinical trial has to be documented on the patient’s health records.

### Confidentiality

Patients will be informed about data protection and that data will be pseudonymized and handed over to a third party after being anonymized. Access to encoded data or source documents will only be given to authorized bodies or persons (sponsor, authorized staff, auditors, competent authorities or ethics commission) for validation of data. Also in case of publication, confidentiality of the collected data will be guaranteed. A unique randomization number will identify all subjects. Each investigator will maintain a subject identification list according to the Sponsor’s SOP, which enables the identification of the subjects by withholding information about the subject’s personal data and randomization number. This list will be stored safely (limited access) in the investigator’s site file. The subject’s informed consent, which bears subject’s printed name and signature, will be filed separately in another investigators’ file. Monitors, auditors or the competent Ethics Committee will have access to personal data but copying of the identification list or an informed consent is without exception prohibited.

Where required, personal data, and health data in particular, may be handled as follows:

- Held for inspection by the competent ethics committee for monitoring the orderly performance of the study,
- Passed on to the investigators or an authorized party for analysis under a pseudonym.

### Access to data

Following termination of the study, all principle investigators, the sponsor and Baxter will have access to the cleaned data sets.

### Ancillary and post-trial care

No specific post-study arrangements are made and no specific post-study care will be performed after this study. All patients will return to their standard medical care subsequently, as needed. This also applies to subjects who withdraw their consent during the course of the study.

### Dissemination policy

The study results will be published in appropriate international scientific journals, and publishing details will be specified in the clinical study agreement. The final report will follow the main CONSORT guideline as well as its extension to non-inferiority trials.

The study is already registered at the registries EudraCT and ClinicalTrials.gov and the principal investigator will disclose the study results there, according to national/international protocol.

## Discussion

We have identified very few RCTs comparing desflurane to other commonly used anesthetics (sevoflurane, propofol and isoflurane) in patients undergoing general anesthesia with LMA [4]. There were no significant differences regarding the frequency of upper airway complications during the anesthesia or at emergence, though the emergence times in the desflurane group were faster. Due to the small sample sizes of these trials and enormous trial inconsistencies [5], there remains a lack of evidence confirming the results of our previous meta-analysis [4]. Furthermore there was a high risk of performance bias in all trials and only two have precisely described the randomization process [11, 12]. In the present large multicenter RCT, all patients will receive the same dosage of additional agents (for example, lidocaine at induction, the same kind of opioids and no muscle relaxants) in regard on their possible impact on the outcome variables. It is not feasible to blind the attending anesthetist to the intervention group. Therefore, we aim to minimize the performance bias by providing a strict but in clinical routine feasible study protocol for the intervention performance. A second, blinded investigator will assess the pre- and postoperative outcome data to reduce the detection bias to a minimum.

Furthermore, the previous trials analyzed in our meta-analysis [4] mainly included ASA I-II patients. However, ASA III patients would also benefit from the LMA, especially in regard to significantly better hemodynamic stability at anesthesia induction and during the emergence. The inclusion of ASA III patients in our RCT will allow us to extend our findings to this patient group. Finally, the recovery on the 1^st^ POD was only assessed in four reviewed trials with different assessment methods [11, 13–15].

We have chosen the PQRS test, as it was also validated for the assessment of later recovery in patients remaining in the hospital as well as via telephone questionnaire once the patient was discharged home [9]. We will perform the PQRS testing on the 1^st^ POD in all patients, either on the ward or via telephone. Another significant advantage of our RCT is that the recruitment and conduction process can work within the usual clinical routine.

### Risk benefit assessment

All drugs used in this trial are anesthetics that have been used in the daily clinical routine for many years. Harms are not expected for any study group. If desflurane enables significant faster emergence from anesthesia, it would have a positive impact on the patient recovery from anesthesia with probably faster discharge times. This would certainly enhance patient comfort and improve cost effectiveness.

## Trial status

Patient recruitment started at the end of February 2015. The predicted study recruiting end-date is September 2015.

## Additional files

Additional file 1:
**Participant timeline.** Schedule of enrollment, interventions and assessments. (PDF 80 kb) Additional file 2:
**Postoperative Quality Recovery Scale.** Assessment Questionnaire. (PDF 8954 kb) Additional file 3:
**Modified Aldrete Score.** (PDF 51 kb) Additional file 4:
**SPIRIT Checklist.** (PDF 106 kb)

## Abbreviations

AE: adverse event
BIS: bispectral index
BMI: body mass index
CE: cough at emergence
CO: intraoperative cough
ECG: electrocardiogram
eCRF: electronic case report form
ETT: endotracheal tubes
GA: general anesthesia
GCP: good clinical practice
HR: heart rate
ICH: International Conference on Harmonisation
ISF: investigator site file
LMA: laryngeal mask airway
LS: laryngospasm
LSE: laryngospasm at emergence
NIBP: noninvasive blood pressure
PACU: ost-anesthesia care unit
PEEP: positive end-expiratory pressure
Pmean: mean pressure
Ppeak: peak pressure
PONV: post-operative nausea and vomiting
PQRS: postoperative quality recovery Scale
SAE: serious adverse event
SNOSE: sequentially numbered, opaque, sealed envelopes
SpO_2_: peripheral oxygen saturation
TLR: time to remove laryngeal mask
TOE: time to open eyes
TRC: time to respond to command
TSB: time to state the date of birth
TSN: time to state the name
1^st^ POD: first postoperative day

## References

[1] Jakobsson J (2012). Desflurane: a clinical update of a third-generation inhaled anaesthetic. Acta Anaesthesiol Scand.

[2] Yu SH, Beirne OR (2010). Laryngeal mask airways have a lower risk of airway complications compared with endotracheal intubation: a systematic review. J Oral Maxillofac Surg.

[3] de Oliveira GS, Girao W, Fitzgerald PC, McCarthy RJ (2013). The effect of sevoflurane versus desflurane on the incidence of upper respiratory morbidity in patients undergoing general anesthesia with a Laryngeal Mask Airway: a meta-analysis of randomized controlled trials. J Clin Anesth.

[4] Stevanovic A, Rossaint R, Fritz HG, Froeba G, Heine J, Puehringer FK (2014). Airway reactions and emergence times in general laryngeal mask airway anaesthesia A meta-analysis. Eur J Anaesthesiol.

[5] Afshari A, Wetterslev J (2015). When may systematic reviews and meta-analyses be considered reliable?. Eur J Anaesthesiol.

[6] Rossaint R, Reyle-Hahn M, Schulte Am Esch J, Scholz J, Scherpereel P, Vallet B (2003). Multicenter randomized comparison of the efficacy and safety of xenon and isoflurane in patients undergoing elective surgery. Anesthesiology.

[7] Blackwelder WC (1982). “Proving the null hypothesis” in clinical trials. Control Clin Trials.

[8] R Core Team. R: A language and environment for statistical computing. R Foundation for Statistical Computing, Vienna, Austria. 2013. http://www.r-project.org. Accessed Nov 2014

[9] Royse CF, Newman S, Chung F, Stygall J, McKay RE, Boldt J (2010). Development and feasibility of a scale to assess postoperative recovery: the post-operative quality recovery scale. Anesthesiology.

[10] Williamson A, Hoggart B (2005). Pain: a review of three commonly used pain rating scales. J Clin Nurs.

[11] De Oliveira GS, Fitzgerald PC, Ahmad S, Marcus RJ, McCarthy RJ (2013). Desflurane/fentanyl compared with sevoflurane/fentanyl on awakening and quality of recovery in outpatient surgery using a laryngeal mask airway: a randomized, double-blinded controlled trial. J Clin Anesth.

[12] Lema FE, Tafur LA, Giraldo C, Delgado MA (2010). Incidence of cough after desflurane and sevoflurane administration through a laryngeal mask: a controlled clinical trial. Rev Esp Anestesiol Reanim.

[13] Gupta A, Kullander M, Ekberg K, Lennmarken C (1996). Anaesthesia for day-care arthroscopy. A comparison between desflurane and isoflurane. Anaesthesia.

[14] Mahmoud NA, Rose DJ, Laurence AS (2001). Desflurane or sevoflurane for gynaecological day-case anaesthesia with spontaneous respiration?. Anaesthesia.

[15] White PF, Tang J, Wender RH, Yumul R, Stokes OJ, Sloninsky A (2009). Desflurane versus sevoflurane for maintenance of outpatient anesthesia: the effect on early versus late recovery and perioperative coughing. Anesth Analg.

