# Supplementary material for: Emergence times and airway reactions in general laryngeal mask airway anesthesia: study protocol for a randomized controlled trial
Source: Trials. 2015 Jul 26;16:316. doi: 10.1186/s13063-015-0855-2 (PMC4515321; doi:10.1186/s13063-015-0855-2)
Supplement: Additional file 1: — Participant timeline. Schedule of enrollment, interventions and assessments. (PDF 80 kb) [file 13063_2015_855_MOESM1_ESM.pdf]

Additional file 1. Participant timeline. Schedule of enrolment, interventions and assessments

	STUDY PERIOD						
	**Enrolment**	**Allocation**	**Post-allocation**				**Close-out**
**TIME POINT**	***A***	***B***	***C***	***T_0***	***D***	***E***	***F***
**ENROLMENT:**							
**Eligibility screen**	X						
**Informed consent**	X						
**Allocation**		X					
**INTERVENTIONS:**							
Desfurane			⟷	⟷			
Sevoflurane			⟷	⟷			
Propofol			⟷	⟷			
**ASSESSMENTS:**							
**Baseline variables**<br>• Age, gender, weight, height, BMI, ASA, smoking, contraceptives<br>• Pre-existing diseases and medical /surgical history<br>• Apfel Score, Baseline PQRS	X	X					
**Intraoperative outcome variables**<br>• BIS, ECG, SpO2, PCO2et, NIBP, Ppeak, Pmean, PEEP<br>• Type of surgery, time-points of anesthesia induction, LMA insertion, study treatments and anesthesia and surgery duration<br>• End-exp. anesthetic concentration/ applied amount of propofol<br>• Total amount of used: catecholamines, remifentanil, piritramide, rescue-propofol, wasted propofol<br>• Frequency of intraoperative (induction/maintenance) coughs and laryngospasm			X				

**<u>Assessing of the time point of cessation of anesthesia</u>**				X			
**<u>Postoperative outcome variables assessed in the operating room</u>**<br>• Frequency of cough at emergence and laryngospasms<br>• Emergence times: time to open eyes, to remove LMA, to respond to command (press hand), to state the date of birth and to state the name<br>• Modified Aldrete score 5 min. after removal of LMA					X		
**<u>Postoperative outcome variables assessed in the Postanesthesia Care Unit (PACU)</u>**<br>• The modified Aldrete scores<br>• Time-point of readiness to be discharged from PACU<br>• PQRS-T40<br>• Visual Analogue Scale (VAS) pain score<br>• Nausea assessed by Verbal Rating Scale (VRS)<br>• Frequency of vomiting<br>• Amount of used piritramide						X	
**<u>Postoperative outcome variables 1st POD</u>**<br>• PQRS-D1							X
