# Supplementary material for: Emergence times and airway reactions in general laryngeal mask airway anesthesia: study protocol for a randomized controlled trial
Source: Trials. 2015 Jul 26;16:316. doi: 10.1186/s13063-015-0855-2 (PMC4515321; doi:10.1186/s13063-015-0855-2)
Supplement: Additional file 2: — Postoperative Quality Recovery Scale. Assessment Questionnaire. (PDF 8954 kb) [file 13063_2015_855_MOESM2_ESM.pdf]

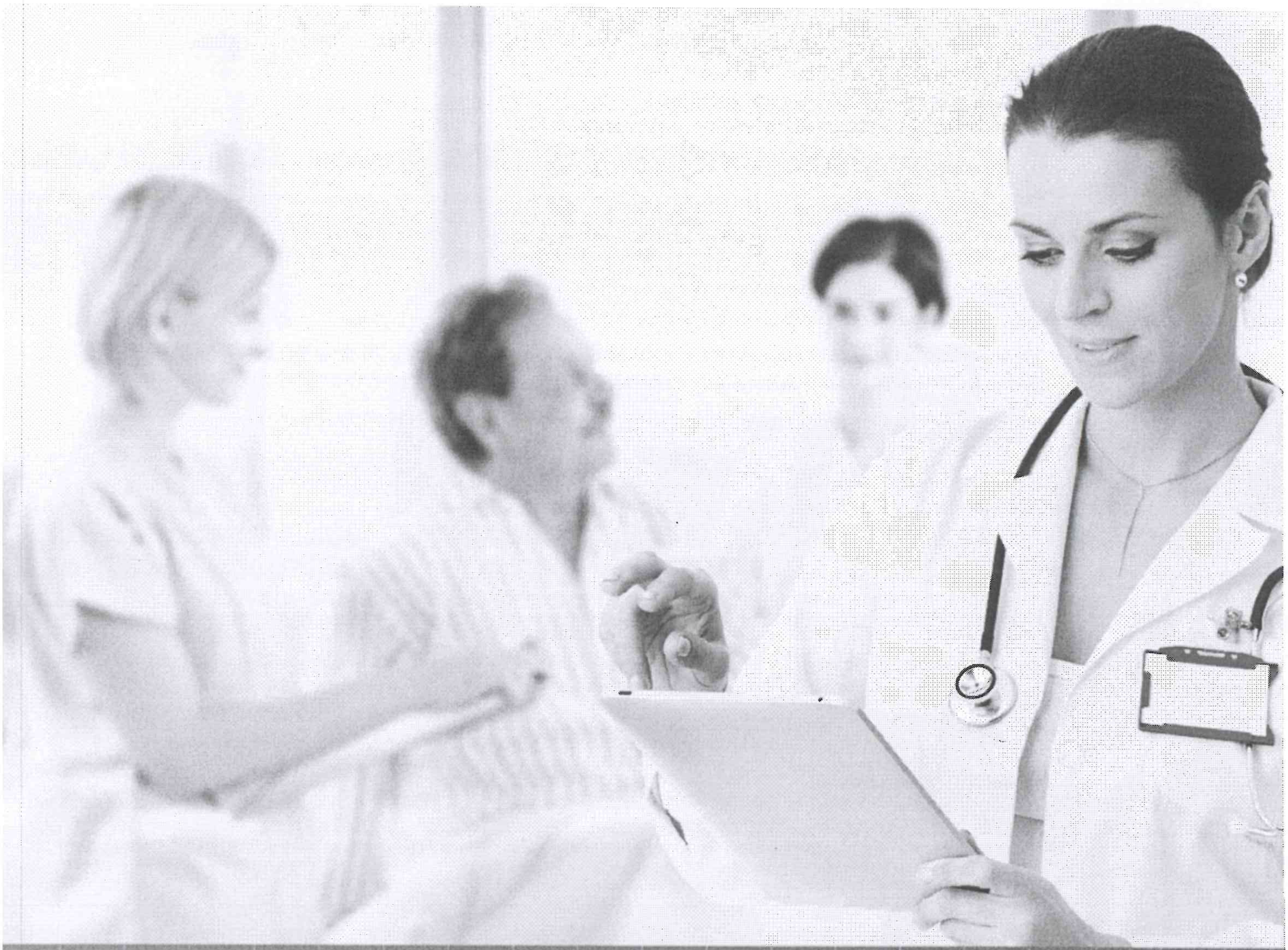

PQRS

Post-operative Quality Recovery Scale

Patient Assessment Questionnaire Booklet

Please complete the information required and conduct the testing according to the instructions provided with each question. A detailed explanation of the tests and instructions are contained separately and provided as part of the training program. The Cognitive Domain testing takes the longest time to complete. A stopwatch should be used to time the completion the executive memory task of the Cognitive Domain. These assessments should be conducted in as quiet an environment as possible given the circumstances. For further information please consult the principal researcher in your facility.

To enter this data onto the online database use the 'Paper-based quick entry' option for this patient.

Pre-surgery (baseline) - Pre-surgery (baseline)

Physiological Factors

P1	Please record the patient's systolic blood pressure	☐ 1 = < 70 or > 180<br>☐ 2 = 70 - 89 or 141 - 180<br>☐ 3 = 90 - 140
P2	Heart Rate<br>Please record the patient's heart rate	☐ 1 = < 35 or > 140<br>☐ 2 = 35 - 44 or 101 - 139<br>☐ 3 = 45 - 100
P3	Temperature<br>Please record the patient's temperature	☐ 1 = < 35 or > 39<br>☐ 2 = 35 - 35.9 or 37.7 - 38.9<br>☐ 3 = 36 - 37.6
P4	Respiration<br>Please record the patient's respiratory rate	☐ 1 = <5 or >30<br>☐ 2 = 5 - 9 or 21 - 30<br>☐ 3 = 10 - 20
P5	Oxygen use to maintain SpO2<br>Please record O2 requirement	☐ 1 = Any SpO2 <90% requiring oxygen as an intervention<br>☐ 2 = Any SpO2 <95% requiring oxygen as an intervention<br>☐ 3 = O2 administered by protocol or not required
P6	Airway<br>Please record the number corresponding to the actual assessment	☐ 1 = Device in situ<br>☐ 2 = Maintenance of airway with support<br>☐ 3 = Self maintenance of airway
P7	Agitation<br>Please record the number corresponding to the actual assessment	☐ 1 = Patient shows severe agitation<br>☐ 2 = Patient shows occasional agitation<br>☐ 3 = Shows no sign of agitation
P8	Consciousness<br>Please record the number corresponding to the actual assessment	☐ 1 = Not rousable<br>☐ 2 = Rousable on auditory or physical stimulation<br>☐ 3 = Fully awake
P9	Please touch your nose or please lift your head?<br>Please record the number corresponding to the actual assessment	☐ 1 = No patient response or purposeless movement<br>☐ 2 = Patient responds purposely but is unable to complete request<br>☐ 3 = Patient follows command completely

Nociceptive Factors

N1	I am going to show you a series of faces and I would like you to indicate which face, number or description most accurately describes your level of pain at the moment?<br>Please show the appropriate face chart to the patient and record the number corresponding to the actual response	☐ 1 = No pain<br>☐ 2 = Mild pain<br>☐ 3 = Moderate pain<br>☐ 4 = Severe pain<br>☐ 5 = Worst possible pain
N2	I am going to show you a series of faces and I would you to indicate which face, number or description most accurately describes your level of feeling nauseous or vomiting at the moment?<br>Please show the appropriate face chart to the patient and record the number corresponding to the actual response	☐ 1 = No nausea, dry retching or vomiting<br>☐ 2 = Mild nausea and no dry retching / vomiting<br>☐ 3 = Moderate nausea and or dry retching / vomiting<br>☐ 4 = Severe nausea and or dry retching / vomiting<br>☐ 5 = Continuous dry retching / vomiting

Emotional Factors

E1	I am going to show you a series of faces and I would like you to indicate which face, number or description most accurately describes to what extent you feel sad, low or depressed at the moment?<br>Please show the appropriate face chart to the patient and record the number corresponding to the actual response	☐ 1 = Not at all depressed / sad<br>☐ 2 = A little depressed / sad<br>☐ 3 = Somewhat depressed / sad<br>☐ 4 = Quite depressed / sad<br>☐ 5 = Extremely depressed / sad
E2	I am going to show you a series of faces and I would like you to indicate which face, number or description most accurately describes to what extent you feel anxious or nervous at the moment?<br>Please show the appropriate face chart to the patient and record the number corresponding to the actual response	☐ 1 = Not at all anxious / nervous<br>☐ 2 = A little anxious / nervous<br>☐ 3 = Somewhat anxious / nervous<br>☐ 4 = Quite anxious / nervous<br>☐ 5 = Extremely anxious / nervous

ADL Factors

A1	**Are you able to stand without assistance?**<br>Please record the number corresponding to the actual assessment	☐ 1 = Not at all<br>☐ 2 = With difficulty<br>☐ 3 = Easily
A2	**Are you able to walk without assistance?**<br>Please record the number corresponding to the actual assessment	☐ 1 = Not at all<br>☐ 2 = With difficulty<br>☐ 3 = Easily
A3	**Are you able to eat or drink without assistance?**<br>Please record the number corresponding to the actual assessment	☐ 1 = Not at all<br>☐ 2 = With difficulty<br>☐ 3 = Easily
A4	**Are you able to dress yourself without assistance?**<br>Please record the number corresponding to the actual assessment	☐ 1 = Not at all<br>☐ 2 = With difficulty<br>☐ 3 = Easily

Cognitive Factors

C1	**Please tell me your name, the city we are in and your date of birth.**<br>Please record the number of correct responses	# Correct responses ☐
C2	**I am going to read you a list of numbers. Listen carefully, then when I am finished, I would like you to repeat them back to me in the same order that I read them. So, for example, if I said 1,2,3, you would say 1,2,3.**<br>Read out the digits given at the rate of one per second. Stop after failure at any point. Please record the item number of the last line correctly recalled.	5, 6 — 1 = ☐<br>1, 6, 4 — 2 = ☐<br>7, 1, 9, 4 — 3 = ☐<br>8, 3, 9, 6, 2 — 4 = ☐<br>5, 2, 8, 7, 9, 4 — 5 = ☐<br>6, 8, 5, 1, 3, 9, 7 — 6 = ☐
C3	**I am going to read you some more numbers, but this time when I stop I would like you to say them in reverse order. So, for example, if I said 1,2,3 you would say 3,2,1.**<br>Read out the digits given at the rate of one per second. Stop after failure at any point. Please record the item number of the last line correctly recalled.	3, 4 — 1 = ☐<br>1, 5 ,9 — 2 = ☐<br>6, 2, 7, 3 — 3 = ☐<br>8, 4, 7, 6, 1 — 4 = ☐<br>9, 2, 4, 7, 1, 3 — 5 = ☐<br>4, 1, 6, 9, 5, 2, 7 — 6 = ☐
C4	**I am going to read out a list of words. Please listen carefully as when I have finished I would like you to repeat back to me as many of the words as you can remember. You can say them in any order and if you are not sure if you have said a word, say it just in case.**<br>Read the words to the patient at about 1 per second. Please record the number of correct responses	**BOOK, FLOWER, TRAIN, RUG, MEADOW, HARP, SALT, FINGER, APPLE, CHIMNEY, BUTTON, KEY, DOG, TREE, RATTLE,**<br># Correct responses ☐

C5	I am going to name a letter and I would like you to state as many words as you can in 30 secs that begin with this letter, try to avoid proper nouns, such as peoples names, names of countries etc, numbers or the same word with a different ending such as long, longer, longish. The letter is "F".<br><br>Time for 30 seconds using a stopwatch and stop patient at this time point. Please record the number of words correctly given in the 30 second time period.	# Correct responses

Early recovery - T0+40m

Physiological Factors		
P1	Please record the patient's systolic blood pressure	☐ 1 = < 70 or > 180<br>☐ 2 = 70 - 89 or 141 - 180<br>☐ 3 = 90 - 140
P2	Heart Rate<br>Please record the patient's heart rate	☐ 1 = < 35 or > 140<br>☐ 2 = 35 - 44 or 101 - 139<br>☐ 3 = 45 - 100
P3	Temperature<br>Please record the patient's temperature	☐ 1 = < 35 or > 39<br>☐ 2 = 35 - 35.9 or 37.7 - 38.9<br>☐ 3 = 36 - 37.6
P4	Respiration<br>Please record the patient's respiratory rate	☐ 1 = <5 or >30<br>☐ 2 = 5 - 9 or 21 - 30<br>☐ 3 = 10 - 20
P5	Oxygen use to maintain SpO2<br>Please record O2 requirement	☐ 1 = Any SpO2 <90% requiring oxygen as an intervention<br>☐ 2 = Any SpO2 <95% requiring oxygen as an intervention<br>☐ 3 = O2 administered by protocol or not required
P6	Airway<br>Please record the number corresponding to the actual assessment	☐ 1 = Device in situ<br>☐ 2 = Maintenance of airway with support<br>☐ 3 = Self maintenance of airway
P7	Agitation<br>Please record the number corresponding to the actual assessment	☐ 1 = Patient shows severe agitation<br>☐ 2 = Patient shows occasional agitation<br>☐ 3 = Shows no sign of agitation
P8	Consciousness<br>Please record the number corresponding to the actual assessment	☐ 1 = Not rousable<br>☐ 2 = Rousable on auditory or physical stimulation<br>☐ 3 = Fully awake
P9	Please touch your nose or please lift your head?<br>Please record the number corresponding to the actual assessment	☐ 1 = No patient response or purposeless movement<br>☐ 2 = Patient responds purposely but is unable to complete request<br>☐ 3 = Patient follows command completely

Nociceptive Factors

N1	**I am going to show you a series of faces and I would like you to indicate which face, number or description most accurately describes your level of pain at the moment?**<br>Please show the appropriate face chart to the patient and record the number corresponding to the actual response	☐ 1 = No pain<br>☐ 2 = Mild pain<br>☐ 3 = Moderate pain<br>☐ 4 = Severe pain<br>☐ 5 = Worst possible pain
N2	**I am going to show you a series of faces and I would you to indicate which face, number or description most accurately describes your level of feeling nauseous or vomiting at the moment?**<br>Please show the appropriate face chart to the patient and record the number corresponding to the actual response	☐ 1 = No nausea, dry retching or vomiting<br>☐ 2 = Mild nausea and no dry retching / vomiting<br>☐ 3 = Moderate nausea and or dry retching / vomiting<br>☐ 4 = Severe nausea and or dry retching / vomiting<br>☐ 5 = Continuous dry retching / vomiting

Emotional Factors

E1	**I am going to show you a series of faces and I would like you to indicate which face, number or description most accurately describes to what extent you feel sad, low or depressed at the moment?**<br>Please show the appropriate face chart to the patient and record the number corresponding to the actual response	☐ 1 = Not at all depressed / sad<br>☐ 2 = A little depressed / sad<br>☐ 3 = Somewhat depressed / sad<br>☐ 4 = Quite depressed / sad<br>☐ 5 = Extremely depressed / sad
E2	**I am going to show you a series of faces and I would like you to indicate which face, number or description most accurately describes to what extent you feel anxious or nervous at the moment?**<br>Please show the appropriate face chart to the patient and record the number corresponding to the actual response	☐ 1 = Not at all anxious / nervous<br>☐ 2 = A little anxious / nervous<br>☐ 3 = Somewhat anxious / nervous<br>☐ 4 = Quite anxious / nervous<br>☐ 5 = Extremely anxious / nervous

ADL Factors

A1	**Are you able to stand without assistance?**<br>Please record the number corresponding to the actual assessment	☐ 1 = Not at all<br>☐ 2 = With difficulty<br>☐ 3 = Easily
A2	**Are you able to walk without assistance?**<br>Please record the number corresponding to the actual assessment	☐ 1 = Not at all<br>☐ 2 = With difficulty<br>☐ 3 = Easily
A3	**Are you able to eat or drink without assistance?**<br>Please record the number corresponding to the actual assessment	☐ 1 = Not at all<br>☐ 2 = With difficulty<br>☐ 3 = Easily
A4	**Are you able to dress yourself without assistance?**<br>Please record the number corresponding to the actual assessment	☐ 1 = Not at all<br>☐ 2 = With difficulty<br>☐ 3 = Easily

Cognitive Factors

C1	**Please tell me your name, the city we are in and your date of birth.**<br>Please record the number of correct responses	# Correct responses ☐
C2	**I am going to read you a list of numbers. Listen carefully, then when I am finished, I would like you to repeat them back to me in the same order that I read them. So, for example, if I said 1,2,3, you would say 1,2,3.**<br>Read out the digits given at the rate of one per second. Stop after failure at any point. Please record the item number of the last line correctly recalled.	6, 7 — 1 = ☐<br>2, 5, 3 — 2 = ☐<br>6, 3, 8, 2 — 3 = ☐<br>5, 7, 3, 6, 1 — 4 = ☐<br>4, 3, 9, 5, 2, 8 — 5 = ☐<br>1, 7, 9, 5, 3, 2, 4 — 6 = ☐
C3	**I am going to read you some more numbers, but this time when I stop I would like you to say them in reverse order. So, for example, if I said 1,2,3 you would say 3,2,1.**<br>Read out the digits given at the rate of one per second. Stop after failure at any point. Please record the item number of the last line correctly recalled.	5, 6 — 1 = ☐<br>3, 7, 4 — 2 = ☐<br>5, 9, 1, 3 — 3 = ☐<br>7, 6, 8, 2, 4 — 4 = ☐<br>3, 6, 1, 5, 9, 2 — 5 = ☐<br>1, 4, 8, 6, 3, 9, 2 — 6 = ☐
C4	**I am going to read out a list of words. Please listen carefully as when I have finished I would like you to repeat back to me as many of the words as you can remember. You can say them in any order and if you are not sure if you have said a word, say it just in case.**<br>Read the words to the patient at about 1 per second. Please record the number of correct responses	DRUM, CURTAIN, BELL, COFFEE, SCHOOL, PARENT, MOON, GARDEN, HAT, FARMER, NOSE, TURKEY, COLOUR, HOUSE, RIVER,<br># Correct responses ☐

C5	I am going to name a letter and I would like you to state as many words as you can in 30 secs that begin with this letter, try to avoid proper nouns, such as peoples names, names of countries etc, numbers or the same word with a different ending such as long, longer, longish. The letter is "C".<br><br>Time for 30 seconds using a stopwatch and stop patient at this time point. Please record the number of words correctly given in the 30 second time period.	# Correct responses

Late recovery (2) - T0+1d

Nociceptive Factors

N1	**I am going to show you a series of faces and I would like you to indicate which face, number or description most accurately describes your level of pain at the moment?**<br>Please show the appropriate face chart to the patient and record the number corresponding to the actual response	☐ 1 = No pain<br>☐ 2 = Mild pain<br>☐ 3 = Moderate pain<br>☐ 4 = Severe pain<br>☐ 5 = Worst possible pain
N2	**I am going to show you a series of faces and I would you to indicate which face, number or description most accurately describes your level of feeling nauseous or vomiting at the moment?**<br>Please show the appropriate face chart to the patient and record the number corresponding to the actual response	☐ 1 = No nausea, dry retching or vomiting<br>☐ 2 = Mild nausea and no dry retching / vomiting<br>☐ 3 = Moderate nausea and or dry retching / vomiting<br>☐ 4 = Severe nausea and or dry retching / vomiting<br>☐ 5 = Continuous dry retching / vomiting

Emotional Factors

E1	**I am going to show you a series of faces and I would like you to indicate which face, number or description most accurately describes to what extent you feel sad, low or depressed at the moment?**<br>Please show the appropriate face chart to the patient and record the number corresponding to the actual response	☐ 1 = Not at all depressed / sad<br>☐ 2 = A little depressed / sad<br>☐ 3 = Somewhat depressed / sad<br>☐ 4 = Quite depressed / sad<br>☐ 5 = Extremely depressed / sad
E2	**I am going to show you a series of faces and I would like you to indicate which face, number or description most accurately describes to what extent you feel anxious or nervous at the moment?**<br>Please show the appropriate face chart to the patient and record the number corresponding to the actual response	☐ 1 = Not at all anxious / nervous<br>☐ 2 = A little anxious / nervous<br>☐ 3 = Somewhat anxious / nervous<br>☐ 4 = Quite anxious / nervous<br>☐ 5 = Extremely anxious / nervous

ADL Factors

A1	**Are you able to stand without assistance?**<br>Please record the number corresponding to the actual assessment	☐ 1 = Not at all<br>☐ 2 = With difficulty<br>☐ 3 = Easily
A2	**Are you able to walk without assistance?**<br>Please record the number corresponding to the actual assessment	☐ 1 = Not at all<br>☐ 2 = With difficulty<br>☐ 3 = Easily
A3	**Are you able to eat or drink without assistance?**<br>Please record the number corresponding to the actual assessment	☐ 1 = Not at all<br>☐ 2 = With difficulty<br>☐ 3 = Easily
A4	**Are you able to dress yourself without assistance?**<br>Please record the number corresponding to the actual assessment	☐ 1 = Not at all<br>☐ 2 = With difficulty<br>☐ 3 = Easily

Cognitive Factors

C1	**Please tell me your name, the city we are in and your date of birth.**<br>Please record the number of correct responses	# Correct responses ☐
C2	**I am going to read you a list of numbers. Listen carefully, then when I am finished, I would like you to repeat them back to me in the same order that I read them. So, for example, if I said 1,2,3, you would say 1,2,3.**<br>Read out the digits given at the rate of one per second. Stop after failure at any point. Please record the item number of the last line correctly recalled.	5, 6 — 1 = ☐<br>1, 6, 4 — 2 = ☐<br>7, 1, 9, 4 — 3 = ☐<br>8, 3, 9, 6, 2 — 4 = ☐<br>5, 2, 8, 7, 9, 4 — 5 = ☐<br>6, 8, 5, 1, 3, 9, 7 — 6 = ☐
C3	**I am going to read you some more numbers, but this time when I stop I would like you to say them in reverse order. So, for example, if I said 1,2,3 you would say 3,2,1.**<br>Read out the digits given at the rate of one per second. Stop after failure at any point. Please record the item number of the last line correctly recalled.	3, 4 — 1 = ☐<br>1, 5 ,9 — 2 = ☐<br>6, 2, 7, 3 — 3 = ☐<br>8, 4, 7, 6, 1 — 4 = ☐<br>9, 2, 4, 7, 1, 3 — 5 = ☐<br>4, 1, 6, 9, 5, 2, 7 — 6 = ☐
C4	**I am going to read out a list of words. Please listen carefully as when I have finished I would like you to repeat back to me as many of the words as you can remember. You can say them in any order and if you are not sure if you have said a word, say it just in case.**<br>Read the words to the patient at about 1 per second. Please record the number of correct responses	**BOOK, FLOWER, TRAIN, RUG, MEADOW, HARP, SALT, FINGER, APPLE, CHIMNEY, BUTTON, KEY, DOG, TREE, RATTLE,**<br># Correct responses ☐

C5	I am going to name a letter and I would like you to state as many words as you can in 30 secs that begin with this letter, try to avoid proper nouns, such as peoples names, names of countries etc, numbers or the same word with a different ending such as long, longer, longish. The letter is "F".<br>Time for 30 seconds using a stopwatch and stop patient at this time point. Please record the number of words correctly given in the 30 second time period.	# Correct responses ☐

Overall Patient Perspective

O1	I am going to show you a series of faces and I would like you to indicate which face, number or description you believe most accurately describes to what extend your surgical procedure has negatively effected your ability to work compared to before your surgery?<br>Please show the appropriate face chart to the patient and record the number corresponding to the actual response	☐ 1 = Not at all impacted<br>☐ 2 = Minimally impacted<br>☐ 3 = Moderately impacted<br>☐ 4 = Severely impacted<br>☐ 5 = Completely impacted
O2	I am going to show you a series of faces and I would like you to indicate which face, number or description you believe most accurately describes to what extent your surgical procedure has negatively effected your ability to undertake daily living activities compared to before your surgery?<br>Please show the appropriate face chart to the patient and record the number corresponding to the actual response	☐ 1 = Not at all impacted<br>☐ 2 = Minimally impacted<br>☐ 3 = Moderately impacted<br>☐ 4 = Severely impacted<br>☐ 5 = Completely impacted
O3	I am going to show you a series of faces and I would like you to indicate which face, number or description you believe most accurately describes to what extend your surical procedure has negatively effected your clarity of thought now compared to before your surgery?<br>Please show the appropriate face chart to the patient and record the number corresponding to the actual response	☐ 1 = Not at all impacted<br>☐ 2 = Minimally impacted<br>☐ 3 = Moderately impacted<br>☐ 4 = Severely impacted<br>☐ 5 = Completely impacted
O4	I am going to show you a series of faces and I would like you to indicate which face, number or description you believe most accurately describes to what extend you were satisfied with the anaesthetic care you received?<br>Please show the appropriate face chart to the patient and record the number corresponding to the actual response	☐ 1 = Totally satisfied<br>☐ 2 = Satisfied<br>☐ 3 = Moderately satisfied<br>☐ 4 = Somewhat satisfied<br>☐ 5 = Not at all satisfied

N1 - PAIN LEVEL CHART

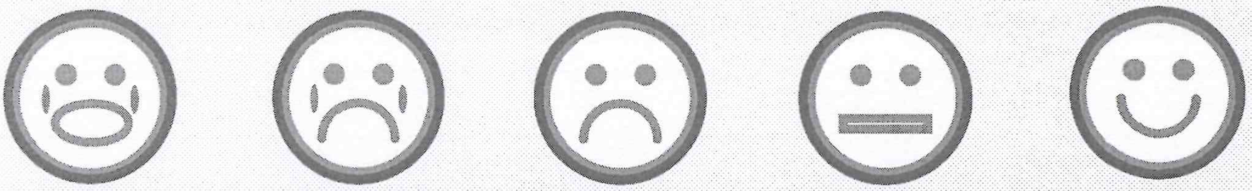

5	4	3	2	1
Worst possible pain	Severe pain	Moderate pain	Mild pain	No pain

N2 - NAUSEA & VOMITING CHART

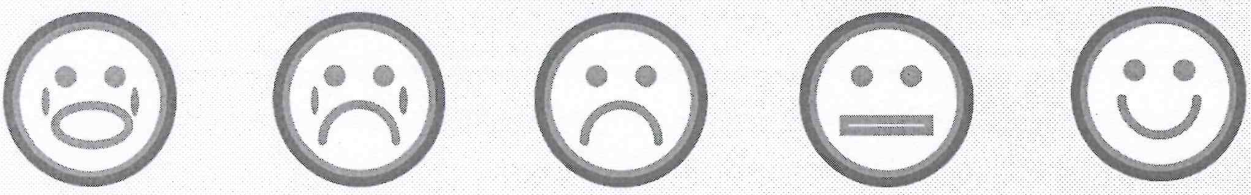

5	4	3	2	1
Continuous dry retching / vomiting	Severe nausea and or dry retching / vomiting	Moderate nausea and or dry retching / vomiting	Mild nausea and no dry retching / vomiting	No nausea, dry retching or/ vomiting

E1 - DEPRESSION / SADNESS CHART

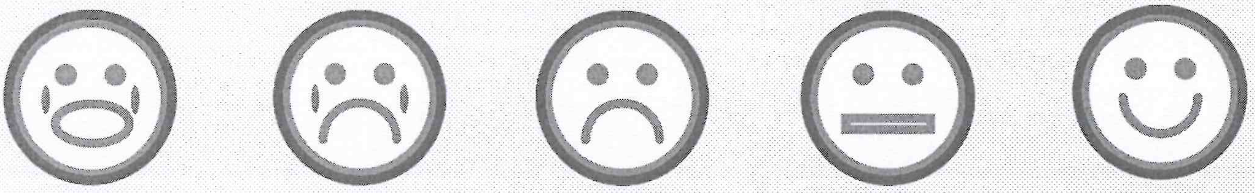

5	4	3	2	1
Extremely depressed / sad	Quite depressed / sad	Somewhat depressed / sad	A little depressed / sad	Not at all depressed / sad

E2 - ANXIETY / NERVOUSNESS CHART

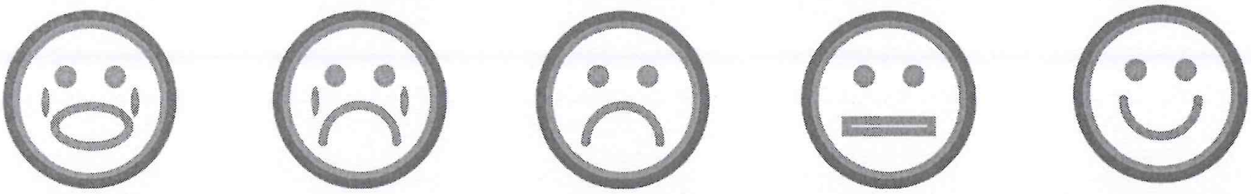

5	4	3	2	1
Extremely anxious / nervous	Quite anxious / nervous	Somewhat anxious / nervous	A little anxious / nervous	Not at all anxious / nervous

01 - EFFECT OF OPERATION ON ABILITY TO WORK CHART CHART

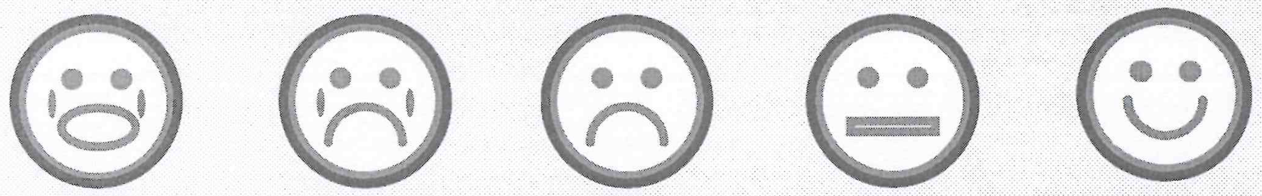

5	4	3	2	1
Completely impacted	Severely impacted	Moderately impacted	Minimally impacted	Not at all impacted

02 - EFFECT OF OPERATION ON UNDERTAKING DAILY LIVING ACTIVITIES CHART

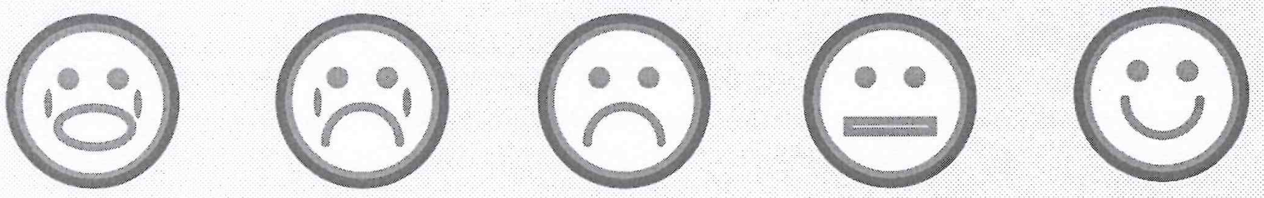

5	4	3	2	1
Completely impacted	Severely impacted	Moderately impacted	Minimally impacted	Not at all impacted

03 - EFFECT OF OPERATION ON CLARITY OF THINKING CHART

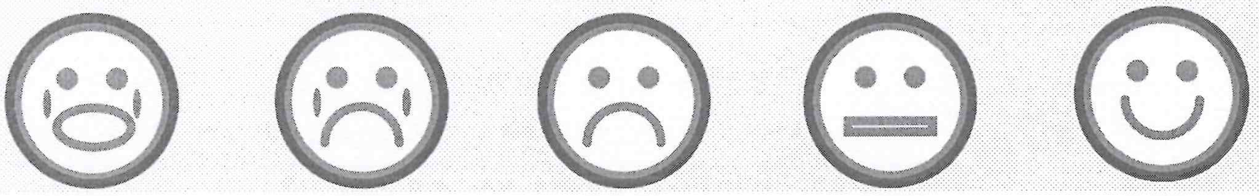

5	4	3	2	1
Completely impacted	Severely impacted	Moderately impacted	Minimally impacted	Not at all impacted

04 - SATISFACTION WITH ANAESTHETIC CARE CHART

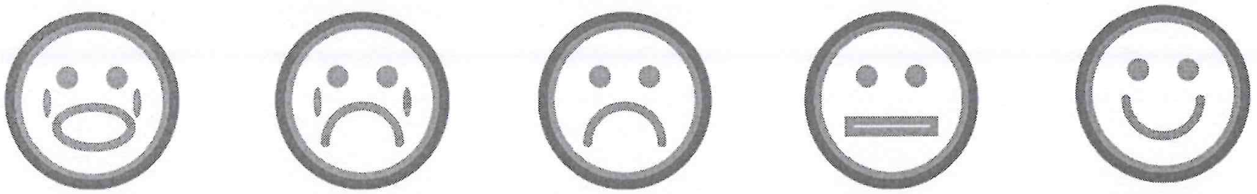

5	4	3	2	1
Not at all satisfied	Somewhat satisfied	Moderately satisfied	Satisfied	Totally satisfied
