# Supplementary material for: Emergence times and airway reactions in general laryngeal mask airway anesthesia: study protocol for a randomized controlled trial
Source: Trials. 2015 Jul 26;16:316. doi: 10.1186/s13063-015-0855-2 (PMC4515321; doi:10.1186/s13063-015-0855-2)
Supplement: Additional file 3: — Modified Aldrete Score. (PDF 51 kb) [file 13063_2015_855_MOESM3_ESM.pdf]

Additional file 3. Modified Aldrete Score

ALDRETE SCORE		VALUE	VALUE	VALUE	VALUE	VALUE
Able to move 4 extremities voluntarily or on command = 2<br>Able to move 2 extremities voluntarily or on command = 1<br>Able to move 0 extremities voluntarily or on command = 0	ACTIVITY					
Able to deep breathe and cough freely = 2<br>Dyspnea or limited breathing = 1<br>Apneic = 0	RESPIRATION					
BP +/- 20% of Preanesthetic level = 2<br>BP +/- 20-50% of Preanesthetic level = 1<br>BP +/- 50% of Preanesthetic level = 0	CIRCULATION					
Fully Awake = 2<br>Arousable on calling = 1<br>Not responding = 0	CONSCIOUSNESS					
Pink = 2<br>Pale, dusky blotchy, jaundiced, other = 1<br>Cyanotic = 0	COLOR					
	**TOTAL**					
DOCUMENTATION DATE / TIME and DISCHARGE to	DATE:<br>TIME:<br>Discharge to	_ _._ _._ _<br>_ _:_ _<br>________	_ _._ _._ _<br>_ _:_ _<br>________	_ _._ _._ _<br>_ _:_ _<br>________	_ _._ _._ _<br>_ _:_ _<br>________	_ _._ _._ _<br>_ _:_ _<br>________
